# Supplementary material for: Acoustic frequency atomic spin oscillator in the quantum regime
Source: Nat Commun. 2023 Oct 12;14:6396. doi: 10.1038/s41467-023-42059-y (PMC10570288; doi:10.1038/s41467-023-42059-y)
Supplement: Supplementary file 1 — Supplementary Information [file 41467_2023_42059_MOESM1_ESM.pdf]

# Supplementary information for “Acoustic frequency atomic spin oscillator in the quantum regime”

## SUPPLEMENTARY NOTE 1 - THE ATOMIC CELL AND THE DRIVE MAGNETIC FIELD

Here is the detailed information for atomic experimental setup:

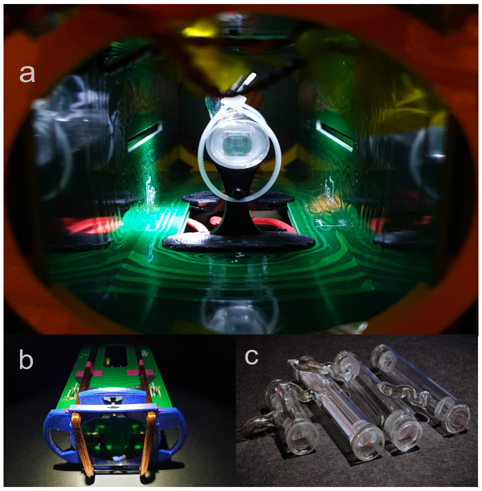


**Supplementary Figure** 1. **Atomic experimental setup.** [**a**]: The cesium vapor cell is surrounded by a specially-designed coil system, which is further protected by a 5-layer magnetic shield. [**b**]: The coil system comprises a combination of rectanglar coils, which produce a concave magnetic field for coarse tuning, and PCBs coils that generate convex and linear-gradient fields for fine tuning. Together, they produce a homogeneous bias magnetic field along the transverse axes of the vapor cell, with an inhomogeneity of less than 1 ‰. [**c**]: The collective atomic spins used in our experiment are confined within a rectangular channel with inner walls coated with C30+ antire-relaxtion material. By utilizing different fabricated cell cross sections, ranging from $(1 \times 1)$ to $(5 \times 5\,\mathrm{mm}^2)$, we could control the dark decoherence rate $\gamma_0$ from 100 to 6 Hz and investigate probe power broadening $\gamma_{s,p}$ given power settings. For this study, a $2 \times 2mm^2$ cell was selected to optimize the balance between the quantum cooperitivity ( $\Gamma_s, \gamma_{s,p}, \gamma_{s0}$) and minimal coupling to environment noise. This allow us to maintain quantum noise limited performance at low acoustic frequency range.

## SUPPLEMENTARY NOTE 2 - VIRTUAL SHIFT OF RESONANCE FREQUENCY

Input-output relations for light quadrature probing atomic ensemble in the QND regime and in the approximation $\Omega \sim |\Omega_S| \gg \gamma_S$ [1]:

$$\begin{bmatrix}\hat{X}_{L,out}\\ \hat{P}_{L,out}\end{bmatrix} = \left\{\begin{bmatrix}1 & 0\\ 0 & 1\end{bmatrix} + 2\Gamma_S \begin{bmatrix}0 & 0\\ \chi_S & 0\end{bmatrix}\right\} \begin{bmatrix}\hat{X}_{L,in}\\ \hat{P}_{L,in}\end{bmatrix} + \sqrt{\Gamma_S\gamma_S}\begin{bmatrix}0 & 0\\ -i\chi_S & \chi_S\end{bmatrix}\begin{bmatrix}\hat{\zeta}_X\\ \hat{\zeta}_P,\end{bmatrix} \tag{1}$$

where $\hat{\zeta}_X$ and $\hat{\zeta}_P$ are effective stochastic Langevin forces. Eq. (1) results in the following expression for arbitrary detection quadrature $\hat{Q}_{L,out}(\phi)$=$\hat{P}_{L,out}\cos(\phi)$+$\hat{X}_{L,out}\sin(\phi)$:

$$\hat{Q}_{L,out}(\phi) = \hat{Q}_{L,in}(\phi) + 2\Gamma_S\chi_S\cos(\phi)\hat{X}_{L,in} + \sqrt{2\Gamma_S\gamma_S}\chi_S\cos(\phi)\hat{\zeta}. \tag{2}$$

where $\hat{\zeta} = (-i\hat{\zeta}_X+\hat{\zeta}_P)/\sqrt{2}$ with uncorrelated $\hat{\zeta}_X$ and $\hat{\zeta}_P$. Eq. (2) is then re-written in the new basis $[\hat{Q}_L(\phi), \hat{Q}_L(\phi_\perp)]^T = \boldsymbol{R}(\phi)[\hat{X}_L, \hat{P}_L]^T$ (where $\boldsymbol{R}(\phi)$ is the rotation matrix):

$$\hat{Q}_{L,out}(\phi) = [1 + \chi_S\Gamma_S\sin(2\phi)]\,\hat{Q}_{L,in}(\phi) + 2\chi_S\Gamma_S\cos^2(\phi)\hat{Q}_{L,in}(\phi_\perp) + \sqrt{2\Gamma_S\gamma_S}\chi_S\cos(\phi)\hat{\zeta}. \tag{3}$$

The spectrum of $\hat{Q}_{L,out}(\phi)$ is given by

$$S_S|_{\hat{Q}_L(\phi)} = 1 + 4\Gamma_S^2 \left|\chi_S\right|^2 \cos^2(\phi) + 2\Gamma_S \mathrm{Re}\left[\chi_S\right] \sin(2\phi) + 8\gamma_S\Gamma_S \left|\chi_S\right|^2 S_\zeta \cos^2(\phi), \tag{4}$$

where $S_\zeta$ is the spectrum of $\hat{\zeta}$ and the the input probe light $\left\{\hat{X}_{L,in}, \hat{P}_{L,in}\right\}$ is in the vacuum state. Eq. (4) coincides with the model of the spin noise in the main text, when the broadband noise is absent and the detection efficiency equals unity. We then factor out the Fourier-frequency-dependent response of the oscillator to the thermal force $N_{TN} = \sqrt{2\Gamma_S\gamma_S}\chi_S \cos(\phi)$ on the right-hand side of Eq. (3) and present it in the form $\hat{Q}_{L,out} = N_{TN}\left[\hat{\zeta} + \hat{f}_{LN}\right]$, where we introduced the renormalized light force driving the spin oscillator:

$$\hat{f}_{LN} = \frac{\chi_S^{-1} + \Gamma_S \sin(2\phi)}{\sqrt{2\Gamma_S\gamma_S}\cos(\phi)}\hat{Q}_{L,in}(\phi) + \sqrt{\frac{2\Gamma_S}{\gamma_S}}\cos(\phi)\hat{Q}_{L,in}(\phi_\perp). \tag{5}$$

The tuning of the detection phase $\phi$ leads to the following transformation of the susceptibility function

$$\tilde{\chi}_S^{-1}(\Omega) = \frac{\Omega_S^2 - \Omega^2 - i\gamma_S\Omega}{\Omega_S} + \Gamma_S \sin(2\phi). \tag{6}$$

In particular, the shifted effective frequency $\tilde{\Omega}_S = \Omega_S\sqrt{1 + \Gamma_S \sin(2\phi)/\Omega_S}$ is accompanied by a reduction of the effective readout rate $\tilde{\Gamma}_S = \Gamma_S \cos^2(\phi)$.

To observe the effective shift of the atomic resonance frequency, one can extract the (symmetrized) spectrum $\langle \hat{f}_{LN}^\dagger \hat{f}_{LN} + \hat{f}_{LN}\hat{f}_{LN}^\dagger\rangle/2$ of the light force from experimental data. We implement the procedure for the spin oscillator in the upper audioband ($|\Omega_S|/(2\pi) = 18$ kHz) and demonstrate the frequency downshift $|\Delta\Omega_S|/(2\pi) \approx 2.1$ kHz. In the frequency range $\lesssim 3$ kHz, the model Eq. (4) no longer accurately describes the noise budget, due to the more pronounced impact of DC noise. This prevents the demonstration of a quantum-limited spin oscillator with effective frequency in this range in the present experimental setup.

---

[1] Thomas, R. Optical spin-mechanics quantum interface: entanglement and back-action evasion. PhD dissertation, University of Copenhagen (2020).
